# Mechanisms Linking Olfactory Impairment and Risk of Mortality

**DOI:** 10.3389/fnins.2020.00140

**Published:** 2020-02-21

**Authors:** Victoria Van Regemorter, Thomas Hummel, Flora Rosenzweig, André Mouraux, Philippe Rombaux, Caroline Huart

**Affiliations:** ^1^Department of Anesthesiology, Cliniques Universitaires Saint-Luc, Brussels, Belgium; ^2^Institute of Neuroscience, Université catholique de Louvain, Brussels, Belgium; ^3^Smell and Taste Clinic, Department of Otorhinolaryngology, TU Dresden, Dresden, Germany; ^4^Department of Otorhinolaryngology, Cliniques Universitaires Saint-Luc, Brussels, Belgium

**Keywords:** olfactory impairment, mortality, neurodegenerative diseases, cognitive function, plasticity, aging

## Abstract

Olfaction is a sense involved in a complex set of tasks, influencing eating behavior, increasing awareness of environmental hazards and affecting social communication. Surprisingly, smell disorders are very frequent, especially in the elderly population. Several recent studies conducted mostly in older subjects have demonstrated a strong association between olfactory impairment and overall mortality risk, with anosmia being even more predictive of 5 years mortality risk than cardiovascular disease. Presently, the underlying pathophysiology linking olfactory impairment to mortality remains unknown and only putative mechanisms are suggested. This review aims to examine the link between olfactory impairment and mortality and to discuss existing ideas on underlying existing mechanisms including, (1) the effect of olfactory loss on nutrition, life-threatening situations and social interactions, (2) associated neurodegenerative diseases, (3) accelerated brain aging, and (4) reflection of general health status being reflected in olfactory function.

## Introduction

Olfactory impairment (OI) is known to occur during the process of aging (odds ratio [OR] = 1.55 for every 5-year increase in age) (Schubert et al., 2012). It is estimated that more than 50% of the population aged between 65 and 80 years old exhibit OI, increasing to 75% above 80 years old (Doty and Kamath, 2014). Several hypotheses are proposed to explain this age-related decline of olfaction.

First, the olfactory nerve, originating from the nasal fossa, is the only cranial nerve directly exposed to the environment, which makes it vulnerable to exposure to toxins, infection, trauma, and airborne pollutants (Huart et al., 2013a, b; Ajmani et al., 2016a, b).

Second, age-related alteration of physiological processes and structural changes within the nose, olfactory epithelium, olfactory bulb and higher brain regions seem to contribute greatly to this deterioration (Cerf-Ducastel and Murphy, 2009; Doty and Kamath, 2014; Attems et al., 2015; Gunzer, 2017; Doty, 2018; Marin et al., 2018).

Third, brain aging or environmental exposure could reduce the cellular regeneration found at the different levels of the olfactory system (Huart et al., 2019).

Fourth, genetic factors could also be involved. For example, it has been shown that carriers of val/val genotype of the brain-derived neurotrophic factor (BDNF) val66met polymorphism present a marked aging-associated decline in olfactory function (Hedner et al., 2010). Apolipoprotein E (ApoE) ε4 allele carriers seem to experience greater olfactory decline than the non-carriers (Wang et al., 2002). ApoE may also contribute to neuronal regenerative processes and to the development of neurodegenerative diseases. Interestingly, the combination of ApoE ε4 allele and OI in a non-demented elderly population predicts a larger decline in global cognitive function (Borenstein Graves et al., 1999; Olofsson et al., 2009). As for cognitively impaired individuals, ApoE ε4 allele carriers also show a more significant decline in cognitive abilities as well as in odor identification (Wang et al., 2002). However, a twin study suggests low heritability coefficients regarding olfactory function (Doty et al., 2011).

Fifth, age-related OI could reflect early involvement of olfactory-related brain areas by the neuropathological processes associated with neurodegenerative diseases, such as Alzheimer’s disease (AD) and Parkinson’s disease (PD). Even if the exact mechanisms underlying OI in AD and PD are not completely understood, olfactory brain structures are affected early in their courses (Braak and Del Tredici, 2017; Marin et al., 2018) and OI precedes clinical diagnosis. Regarding AD, OI may already be present in patients with mild cognitive impairment (MCI, cognitive dysfunction exceeding normal “age-related” decline, yet not meeting the criteria for dementia) (Sanford, 2017). It is known that approximately 70% of MCI patients will ultimately convert to AD (Gauthier et al., 2006). Yet, recent data converge toward the idea that olfactory-impaired MCI patients are more prone to develop AD than those without OI (Conti et al., 2013; Devanand, 2016; Adams et al., 2018; Jung et al., 2019). Overall, these findings make OI a potential early predictor for AD development. PD is also marked by early OI, which typically precedes motor symptoms by at least 5 years and thus can be used as a biomarker for the diagnosis of PD (Berardelli et al., 2013; Marin et al., 2018; Haehner et al., 2019). Moreover, a 7-year prospective study conducted in newly diagnosed PD patients reported that hyposmia at diagnosis of PD is associated with further development of cognitive deficits, making OI a predictor of dementia in this setting (Gjerde et al., 2018).

Sixth, OI has been shown to accompany a variety of diseases. In addition to the link to neurodegenerative diseases, OI has been reported to occur in schizophrenia, epilepsy, systemic and endocrine diseases (e.g., hypothyroidism, type 2 diabetes mellitus), chronic kidney or liver failure (Landis et al., 2004; Hummel et al., 2011; Huart et al., 2013a). Of note, a recent study involving type 2 diabetic patients demonstrated not only increased OI in this population compared to controls but also diminished abilities in specific cognitive tests. Besides, once again, they found a strong association between OI and specific memory impairment in this population (Yulug et al., 2019). As for smoking, which is known to interfere with olfactory performance, the effect is equivocal or weak and mostly seen in long-standing heavy smokers (Mackay-Sim et al., 2006). Alcohol’s abuse negative effects on olfaction could be related to alterations in brain areas involved in olfactory processing (Schubert et al., 2012). Higher probability of comorbidities with age comes along with the associated polypharmacy. Despite little evidence-based data, it is commonly thought that medication may affect taste and smell functions (Hummel et al., 2011). Drugs are thought to interact with many different molecular targets of the olfactory pathway from the olfactory receptors to central processes (Lötsch et al., 2012, 2015a,b). A long list of medications might be involved in smell loss, including antibiotics, antidepressant and antipsychotic drugs, antihypertensive drugs (e.g., calcium channel blockers), opioids, sildenafil (Doty and Bromley, 2004; Hummel et al., 2011). However, it is difficult to distinguish drug-induced OI from the effect of the underlying medical issue for which the drug is taken. Landis et al. (2004) did not find a correlation between olfactory function and the number of drugs taken. Nevertheless, a recent study showed a significant correlation between the number of drugs taken and both worse olfactory threshold function and, interestingly, worse MMSE scores (Ottaviano et al., 2018). The authors justified this finding by the fact that these parameters are usually age-related. However, they did not control for comorbidities, which in fact could have explained in part these results.

## Summary of Existing Studies Linking Olfactory Impairment and Mortality

Until now, seven longitudinal studies have been published on the relationship between olfactory function at baseline and risk of mortality (Wilson et al., 2011; Gopinath et al., 2012; Pinto et al., 2014; Devanand et al., 2015; Schubert et al., 2016; Ekström et al., 2017; Liu et al., 2019). Demographics and methods are summarized in Table 1. Statistics and main results are summarized in Table 2. We deliberately decided to include only studies with psychophysical assessment of olfactory function using well-known validated identification tests since self-assessment of olfactory function is not reliable (Landis et al., 2003; Lötsch and Hummel, 2019; Oleszkiewicz and Hummel, 2019; Oleszkiewicz et al., in press). Together, these studies included a total of 13,366 individuals, male and female, aged 40 and older, with older people representing the majority. In regard to age groups, results vary between studies. Some found a stronger OI-mortality link in older subjects, while others demonstrated a slightly stronger relationship in middle-aged groups (Gopinath et al., 2012; Ekström et al., 2017; Liu et al., 2019). This latter finding may be due to the increased prevalence of anosmia with advancing age. Length of follow-up varied according to studies, mainly ranging from 4–5 years up to 10–13 years. Exclusion criteria, mostly including dementia or neurologic diseases, were found in four studies and are discussed below.

**TABLE 1:** Studies linking olfactory impairment and mortality – Demographic data and methods.

References	Sample size (n)	Length of follow-up	Mean age, age range	Exclusion criteria	Olfactory test
Wilson et al., 2011	1162	Mean of 4.2 years, range: 0–9	79.7 years, no data	Diagnosis of dementia or PD	Brief Smell Identification Test (BSIT) (12 points score)
Gopinath et al., 2012	1636	5 years	Presence of OI: 77.2 years, no data Absence of OI: 72.1 years, no data	None	San Diego Odor Identification Test (SDOIT) (8 points score)
Pinto et al., 2014	2918	5 years	68 years, 57–85	None	Validated modified score using items from the Sniffin’ Sticks identification test (5 points score)
Devanand et al., 2015	1169	Mean of 4.1 years, range 0–9.8	80.4 years, ≥65	Clinical stroke, PD, atypical Parkinsonian syndrome diagnoses, schizophrenia, and other psychotic disorders	University of Pennsylvania Smell Identification Test (UPSIT) (40 points score)
Schubert et al., 2016	2418	Mean of 12.8 years, maximum 17 years	69 years, 53–97	None	San Diego Odor Identification Test (SDOIT) (8 points score)
Ekström et al., 2017	1774	10 years	63.5 years, 40–90	Individuals who met criteria for dementia	Modified version of the Scandinavian Odor-Identification test (SOIT) (13 points score)
Liu et al., 2019	2289	13 years	75.6 years, 71–82	Difficulty in walking a quarter mile, climbing 10 steps or performing activities of daily living, active cancer treatment in the previous 3 years	Brief Smell Identification Test (BSIT) (12 points score)

*PD, Parkinson’s disease; OI, olfactory impairment.*

**TABLE 2:** Studies linking olfactory impairment and mortality – Statistics and results.

References	Statistical analyses	Covariates	Main results (according to adjustment for covariates)	
Wilson et al., 2011	Cox proportional hazard models	Age, sex, education	HR = 0.94 (95%CI: 0.90, 0.98)	HR predicting mortality for each additional correct answer on the BSIT score.
		Boston Naming Test	HR = 0.94 (95%CI: 0.90, 0.99)	
		Katz scale, cardiovascular risk factors and conditions	HR = 0.95 (95%CI: 0.90, 0.99)	
		Cognitive, social and physical activity, depressive symptoms	HR = 0.95 (95%CI: 0.91, 0.99)	
Gopinath et al., 2012	Cox proportional hazard models – Multivariate logistic regression	Age, sex	HR = 1.99 (95%CI: 1.42, 2.80)	HR predicting mortality for moderate OI (SDOIT ≤ 3).
		BMI, systolic blood pressure, current smoking status, alcohol consumption, poor self-rated health, visual impairment, hypertension, diabetes, history of cancer, angina, stroke, acute myocardial infarction	HR = 2.04 (95%CI: 1.41, 2.95)	
		Serum total cholesterol	HR = 1.68 (95%CI: 1.10, 2.56)	
		Cognitive impairment	HR = 1.51 (95%CI: 0.96, 2.38)	
Pinto et al., 2014	Multivariate logistic regression	None	OR = 5.85 (95%CI: 3.76, 9.10)	OR predicting mortality for anosmic subjects (4–5 errors on the Sniffin’ Sticks modified score) compared to controls.
		Age, sex, education	OR = 3.24 (95%CI: 1.99, 5.28)	
		Age, sex, education, comorbidity index	OR = 3.41 (95%CI: 2.06, 5.64)	
		Age, sex, education, cardiovascular diseases, cancer, lung disease, stroke, diabetes, liver damage	OR = 3.37 (95%CI: 2.04, 5.57)	
Devanand et al., 2015	Cox proportional hazard models – Multivariate logistic regression	None	HR = 1.068 (95%CI: 1.053, 1.083)	HR predicting mortality for each additional error on the UPSIT score.
		Age, education, race/ethnicity, gender, spanish language, comorbidity index, dementia, depression, head injury, alcohol abuse, BMI, smoking, hearing/vision impairment	HR = 1.049 (95%CI: 1.030, 1.068)	
Schubert et al., 2016	Cox proportional hazard models – Multivariate logistic regression	Age, sex	HR = 1.46 (95%CI: 1.24, 1.73)	HR predicting mortality for OI (SDOIT ≤ 6).
		Age, sex, education, hypertension, diabetes, cardiovascular disease, cancer, cognitive impairment, frailty, smoking, exercise, BMI, alcohol	HR = 1.29 (95%CI: 1.08, 1.54)	
		Age, sex, education, hypertension, diabetes, cardiovascular disease, cancer, cognitive impairment, frailty, smoking, exercise, BMI, alcohol, IMT, CRP, IL-6	HR = 1.28 (95%CI: 1.07, 1.52)	
Ekström et al., 2017	Cox proportional hazard models – Multivariate logistic regression	None	HR = 0.74 (95%CI: 0.71, 0.77)	HR predicting mortality for each additional correct answer on the SOIT.
		Age, sex	HR = 0.91 (95%CI: 0.87, 0.95)	
		Years of education	HR = 0.91 (95%CI: 0.87, 0.96)	
		Health variables	HR = 0.91 (95%CI: 0.87, 0.97)	
		Cognitive performance	HR = 0.92 (95%CI: 0.87, 0.97)	
		Dementia conversion	HR = 0.92 (95%CI: 0.88, 0.97)	
		Apolipoprotein E ε4	HR = 0.92 (95%CI: 0.87, 0.97)	
Liu et al., 2019	Mediation analyses using risk ratio scale	Age, sex, race, education, BMI, drinking, brisk walking, smoking, self-reported general health status, cardiovascular disease, cancer, diabetes, hypertension, depressive symptoms, chronic kidney disease	RR = 1.46 (95%CI: 1.27, 1.67)	RR predicting mortality for OI (BSIT ≤ 8).

*HR, hazard ratio; CI, confidence interval; BMI, body mass index; BSIT, Brief Smell Identification Test; OI, olfactory impairment; SDOIT, San Diego Odor Identification Test; OR, odds ratio; UPSIT, University of Pennsylvania Smell Identification Test; IMT, carotid intima plus media thickness; CRP, C-reactive protein; IL-6, interleukin-6; SOIT, Scandinavian Odor-Identification Test; RR, risk ratio.*

These studies cannot be directly compared due to methodological differences. However, all yield similar results. Regardless of the statistical method used, the relationship between OI and mortality risk was statistically significant and strong. In addition, the association was found to be dose-dependent, meaning anosmic people have a greater risk of death than hyposmic people.

Unfortunately, while clearly revealing the existence of a link between OI and risk of mortality, these studies leave possible explanations of such a link quite unexplored. Indeed, cause-specific mortality is only explored in one study (Liu et al., 2019) whereas others do not report cause of death. Yet, this parameter could have largely contributed to the understanding of the mechanisms of the OI-mortality relationship. Nevertheless, a large number of covariates were controlled, which certainly adds more data to progress through the current hypotheses. Though being a key feature, the causal involvement of neurodegenerative diseases is still left quite unanswered in these studies.

## Possible Mechanisms Linking Olfactory Impairment and Risk of Mortality

At the moment, the underlying physiopathology linking olfactory impairment to mortality remains unknown. Based on existing evidence and on data from the mortality studies, we will present an overview of current hypotheses.

### Effects of Olfactory Loss on Eating Behavior, Danger Warning and Social Interaction

Olfaction is known to play a role in eating behavior, danger warning and social interaction (Stevenson, 2010). OI will therefore have various consequences on these three human functions, which might directly or indirectly lead to decreased health.

First, malnutrition may be a direct consequence of OI as taste and smell are intrinsically linked. In fact, OI may lead to a decreased ability to enjoy food, decreased appetite and food intake, as well as change in body weight and increased risk for chronic disease. People suffering from OI might either maintain their weight, or eat less, or on the contrary eat more since food would become tasteless; reasons for this remain unclear (Croy et al., 2014). Interestingly, there is growing evidence that the olfactory system could greatly contribute in the regulation of food intake and energy balance, through its effect on sympathetic and parasympathetic tone (Palouzier-Paulignan et al., 2012; Garrison and Knight, 2017; Mortreux et al., 2019). Indeed, receptors to a variety of orexigenic and anorexigenic molecules (i.e., insulin, leptin, ghrelin, neuropeptide Y, nutrient glucose) were found in the olfactory epithelium and in the olfactory bulb (Palouzier-Paulignan et al., 2012; Min and Min, 2018). It was shown that older women with OI had poorer diet quality over 5 years (Gopinath et al., 2016) and that a population of older United States female and male adults displayed low body mass index (BMI) (Dong et al., 2017). Still, there remain controversies in the literature regarding the association between malnutrition and OI in geriatric patients (Gunzer, 2017). Similarly, the relationship between OI and obesity yield contradictory results, some studies demonstrating a correlation (mainly through increased threshold detection of odors (Richardson et al., 2004; Palouzier-Paulignan et al., 2012), while others did not (Gouveri et al., 2014). Some authors suggest that the wide range of both etiologies and endocrinologic consequences of obesity could explain these conflicting findings (Palouzier-Paulignan et al., 2012). Among our seven reviewed studies, only a few measured BMI of the subjects. One study found that OI was associated with decreased BMI (Gopinath et al., 2012). The other showed that subjects who did not enjoy eating or had a low BMI had increased mortality, whereas those who had a high BMI had decreased mortality (Pinto et al., 2014). Weight loss, as a surrogate marker for malnutrition, was also found to contribute to the association between OI and mortality (Liu et al., 2019). Indeed, their mediation analysis demonstrated that weight loss could account for, respectively, 6 and 11% of the 10-year and 13-year higher mortality linked to poor olfaction.

Olfactory impairment might also increase risk of death as a result of not smelling danger signals in the household environment. A United States survey showed that, respectively, 20.3 and 31.3% of adults older than 70 years were not able to identify the warning odors of smoke and natural gas (Hoffman et al., 2016). OI thus exposes to risks associated with cooking accidents, fires or gas leaks and ingestion of spoiled foods or toxic substances (Santos et al., 2004). Anosmic people experience hazardous events 2 to 3 times more often than normosmic subjects (Pence et al., 2014). Although the seven studies did not take these events into consideration for analysis of cause-specific mortality, death rate from unintentional poisoning or fire (not necessarily related to OI) is actually low (1.7–4.7 per 100,000) (Kramarow et al., 2015).

Olfactory function is known to influence quality of life. Notably, people with OI typically suffer from depressive symptoms (Gopinath et al., 2011; Croy et al., 2012, 2014; Sivam et al., 2016). One proposed explanation is that dysfunction or absence of olfactory bulb might result in the dysfunction of various cellular processes and pathways within the hippocampus (Morales-Medina et al., 2017). Some of the mortality studies reported that the subjects who died showed more depressive signs (Wilson et al., 2011; Ekström et al., 2017). However, the association of OI with mortality persisted after controlling for depressive symptoms (Wilson et al., 2011). OI is associated with reduced social network size (Zou et al., 2016), and recent work showed that reduced in-person physical contact with others might partially mediate the link between OI and mortality in females (Leschak and Eisenberger, 2018).

### Associated Neurodegenerative Diseases

At present, OI has become a well-known early biomarker for a broad spectrum of neurodegenerative diseases. Therefore, it is not surprising that neurodegenerative diseases have been investigated as a possible mediator in the relationship between OI and mortality. Despite some conflicting results, evidence is progressively increasing that neurodegenerative diseases might partly explain this relationship.

The majority of the reviewed studies included cognitive function at baseline as a covariate. In three studies, controlling for cognitive performance at baseline did not change the results. Nevertheless, Wilson et al. (2011) excluded patients with a diagnosis of dementia or PD at baseline and used a measure of cognitive activity frequency in leisure time, which seems to weakly reflect cognitive function. Schubert et al. (2016) did not adjust the results exclusively for cognitive function but rather included additional covariates for adjustment, so that the precise impact of cognitive function remains unknown. In addition, Ekström et al. (2017) also specifically addressed the question of dementia conversion and found that the OI-mortality relationship was independent from it. On the contrary, three other studies did show a potential mediator effect of cognitive function on mortality (Pinto et al., 2014; Devanand et al., 2015; Liu et al., 2019). Two found that the association between OI and mortality was lowered when controlling for dementia, yet remained statistically significant. Of note, OI was shown to be associated with future cognitive decline in cognitively intact subjects (Devanand, 2016). Moreover, there is evidence of existing post-mortem markers of neurodegenerative disease in the brain of subjects without previous clinical signs of MCI or AD (Wilson et al., 2009). Therefore, one hypothetical explanation for the absence or weak mediating effect of cognitive function on mortality might be that olfaction is altered while clinical cognitive function is not yet affected and thus undiagnosed. Only Gopinath et al. (2012) came to a non-significant association between OI and mortality risk after adjustment for cognitive impairment. This would suggest a mediating role of cognitive function, although the authors clearly state this finding could be due to weak statistical power. More interestingly, the only analysis of cause-specific mortality showed that the strongest causal association found was between OI and death associated with dementia or PD (Liu et al., 2019). Indeed, their mediation analysis showed that dementia or PD could account for 22% of the higher 10-year mortality linked to poor olfaction. Lastly, Devanand et al. (2015) questions the fact that neurodegenerative disorders lead to excess mortality. However, recent evidence suggests otherwise. In 2014, AD was the sixth leading cause of death in the United States, representing 3.6% of total mortality with an age-adjusted death rate of 25.4 per 100,000 citizens (Taylor et al., 2017). As for PD, a meta-analysis showed a pooled mortality ratio of approximately 1.5 compared to controls (Macleod et al., 2014). Also, mean duration until death varies from 6.9 to 14.3 years. This might also partially explain why dementia conversion does not seem to mediate the OI-mortality link despite a 10-year follow-up (Ekström et al., 2017), since death associated with PD may occur after more than 10 years. PD-related mortality was shown to be significantly increased by the presence of MCI at baseline (Hoogland et al., 2019). This finding supports the idea of a correlation between OI, PD and mortality risk.

Finally, adjusting for ApoE ε4 genotype, associated with cognitive decline and OI (Wang et al., 2002), did not attenuate the association between OI and mortality (Devanand et al., 2015; Ekström et al., 2017).

In conclusion, even if available data remain controversial, there seems to be growing evidence that neurodegenerative diseases, through cognitive dysfunction, may be a potential mediator in the relationship connecting OI to mortality risk.

### Accelerated Brain Aging

One of the special features of the olfactory system is its extraordinary plasticity and continuing neurogenesis through adulthood, at least at the level of the olfactory epithelium (for recent review see Huart et al., 2019). Mechanisms underlying these neural abilities have been largely investigated mainly in rodents; indeed, the paucity of human data remains controversial. Adult neurogenesis is thought to occur both peripherally and centrally, in three different locations. First, proliferating stem cells, located within the olfactory epithelium differentiate into olfactory receptor neurons. Secondly, neural stem cells coming from the walls of the lateral ventricle could migrate following the rostral migratory stream toward the olfactory bulb (OB) to give rise to olfactory interneurons (Curtis et al., 2007). Thirdly, some animal data suggest the presence of progenitor cells lying directly within the OB (Huart et al., 2019). For now, however, all this remains somewhat hypothetical in adult human brains. Yet, clinical findings support the idea of high plasticity of the olfactory system. For instance, it is widely acknowledged that the OB is subject to significant volume changes, as a function of olfactory performance. Indeed, OI is associated with reduced OB volume, while recovery of olfactory function correlates with OB volume (Rombaux et al., 2006a, b; Hummel et al., 2013; Yaldizi et al., 2016; Rottstädt et al., 2018). These volume fluctuations probably depend on bottom-up (Negoias et al., 2017) and top-down processes (Cavazzana et al., 2018). In addition, structural brain modifications could also occur beyond the OB (Reichert and Schöpf, 2018). Finally, olfactory plasticity has also been recently highlighted by olfactory training, which was found to improve significantly olfactory skills regardless of baseline olfactory function (Sorokowska et al., 2017) and to lead to increased cortical thickness of certain brain areas (Al Aïn et al., 2019). Interestingly, olfactory training was also shown to improve verbal function and subjective well-being in older people (Wegener et al., 2018).

Importantly, vision and hearing impairment were not associated with mortality nor did they alter the OI-mortality relationship, suggesting that death could not be explained by sensory loss more broadly (Devanand et al., 2015; Schubert et al., 2016). Still, some previous studies have shown a possible link between vision and/or hearing impairment and mortality, evidence for the impact of vision impairment alone remaining weaker (Fisher et al., 2014; Schubert et al., 2016; Lin et al., 2019). It is also important to underline the fact that all humans do not age equally. That is why physiological age might be more relevant than the chronological age. It may be hypothesized that decline in olfactory function could indicate a more advanced physiological age. Indeed, it was found that idiopathic age-related decline in olfactory function was much smaller in the healthy, non-medicated, non-smoking population (Mackay-Sim et al., 2006). On the contrary, it was shown that taste and smell disorders were associated with an increased risk of frailty in the older population (Somekawa et al., 2017; Harita et al., 2019). Among the mortality studies, controlling for performance in activities of daily living or frailty score did not attenuate the effect (Pinto et al., 2014; Schubert et al., 2016). Global sensory impairment (including the five classical senses: smell, taste, hearing, vision and touch) predicts 5-year mortality, but also major components of physical frailty (e.g., slow gait, weight loss, low activity) (Pinto et al., 2017). The same authors developed the concept that multisensory loss of function could reflect a common underlying aging process (Correia et al., 2016).

To summarize, we hypothesize that OI reflects a decline in brain plasticity, and could be seen more globally as an indicator of lowered physiologic repair function. This could explain, at least partly, the pathway toward increased mortality. Brain aging could make the olfactory system as well as other brain structures more vulnerable and less capable of recovering from insults. This highlights the current need to think in terms of physiological age and frailty status. Whether frailty mediates the relationship between OI and mortality still requires more evidence.

### Reflection of General Poor Health

Olfactory impairment is known to be associated with a wide range of illnesses and is thought to be a marker of poor health. Landis et al. (2004) showed a negative correlation between olfactory function and the number of comorbid conditions. Self-reported poor health status was associated with OI in one study (Liu et al., 2019). However, the association between OI and mortality risk was mostly driven by the subjects with baseline excellent to good health, which might question the idea of OI being merely a marker of poor health. The authors hypothesized either that OI in healthy older people might hide an underneath life-threatening condition or that the cumulative effect of multiple comorbid conditions might outweigh the effect of OI on death. Moreover, half of the analyzed studies adjusted their results for comorbid conditions, with either little or no effect on the OI-mortality link (Pinto et al., 2014; Devanand et al., 2015; Schubert et al., 2016; Ekström et al., 2017). Controlling for smoking or alcohol abuse did not attenuate the link between OI and mortality (Pinto et al., 2014; Devanand et al., 2015). Some authors also suggested that high levels of inflammatory markers (interleukin-6, C-reactive protein) could be associated with frailty, atherosclerosis and also with OI (Schubert et al., 2011; Henkin et al., 2013; Laudisio et al., 2019). However, adjusting for these variables did not attenuate the link between OI and mortality (Schubert et al., 2016). Hence, whether OI is a marker of poor health remains doubtful. The only assertion that can be made is that OI is a strong and independent risk factor for death, regardless of health status.

Since controlling for comorbid conditions had little or no effect on the OI-mortality link, this would then suggest a weak impact of cardiovascular diseases or metabolic disorders (being part of the comorbidity indexes used in the studies).

However, analysis of cause-specific mortality revealed that OI was modestly associated with death from cardiovascular disease, but not from cancer or respiratory diseases (Liu et al., 2019). Furthermore, the association between OI and mortality did not remain significant after adjusting for total serum cholesterol, though potentially explained by reduced statistical power (Gopinath et al., 2012). Physical exercise and use of statins were found to decrease the incidence of OI (Schubert et al., 2011). Indeed, both are known to improve cardiovascular health and to lower the risk of atherosclerosis, which is the main hallmark of cardiovascular diseases. Additionally, statins may also directly provide to the brain (and thereby to the olfactory system) their positive pleiotropic effects on endothelial function, oxidative stress and vascular inflammation. This would be consistent with the reduced risk of OI being specific to statins crossing well the blood-brain barrier in the study from Schubert et al. (2011). However, in a mouse model, administration of atorvastatin (classified as not crossing the blood-brain barrier) could still enhance the recovery of olfactory function, likely through the promotion of cellular proliferation and neural regeneration of the olfactory epithelium (Kim et al., 2010, 2012). Carotid artery intima media thickness (IMT), a biomarker of generalized atherosclerosis, was shown to be associated with a decline in odor identification performance at 5 years, but only before 60 years old (Schubert et al., 2015a, b). Nevertheless, the association between OI and mortality remained statistically significant after adjusting for IMT (Schubert et al., 2016). Also, OI could be associated to the intake of antihypertensive drugs (Doty and Bromley, 2004). Therefore, cardiovascular diseases might be somehow connected to OI. Yet, evidence remains sparse and further studies are needed.

Obesity, insulin resistance and type 2 diabetes mellitus constitute a well-known continuum which represents a major burden in terms of public health and mortality. In rodent obesity models, the olfactory bulb developed insulin resistance which could then be responsible for the disruption of olfactory function (Palouzier-Paulignan et al., 2012). Human data also support the fact that insulin resistance could affect negatively olfactory function. Indeed, a recent study found that older adults with high insulin resistance (quantified by the HOMA-IR test) had an approximately 2-fold increased odds of OI compared with subjects with low insulin resistance (Min and Min, 2018). This could explain, at least in part, the independent association found between type 2 diabetes mellitus (in which insulin resistance represents the key mechanism) and OI (Gopinath et al., 2012; Gouveri et al., 2014; Yulug et al., 2019). OI in type 2 diabetes mellitus might also be due to microvascular injury since OI was mainly found in diabetic patients already suffering from the microvascular complications of this disease (Gouveri et al., 2014). Regarding mortality studies, diabetes mellitus was associated with OI in one study (Gopinath et al., 2012) but was not in another one more recent (Liu et al., 2019). Three studies included diabetes mellitus in their controlled variables, but the correlation between OI and mortality remained unchanged, thus also suggesting a weak impact of diabetes mellitus. Regarding the link of obesity, insulin resistance and type 2 diabetes mellitus with OI, controversies thus persist on many points.

Finally, due to its direct contact to the outside, the olfactory system may be an easy target for environmental insults. Indeed, olfaction is altered by air pollutants and by exposure to a variety of toxic agents (e.g., metallic compounds) (Ajmani et al., 2016a, b; Genter and Doty, 2019). The airborne toxicants may also make their way to the brain directly through the olfactory pathway, leading to neuronal damage. Of note, it was shown that high levels of air pollution could result in neurodegenerative diseases-related pathology within the olfactory bulb (Calderon-Garciduenas et al., 2018). Pollution and toxic exposure have been linked to poor health and might thus constitute a potential mediator in the relationship between OI and mortality (Fuller-Thomson and Fuller-Thomson, 2019).

## Conclusion

Studies agree that OI is an indicator of increased mortality risk in the older population. Several hypothetical mechanisms are suggested, yet the extent to which they contribute to this association remains an open question. To our opinion, the effects of olfactory impairment on eating behavior, danger warning and social interaction do exist, although their importance in this context seems minor. Olfactory impairment and poor health status seem to be linked in some ways, though the exact relationship remains poorly understood. On the contrary, based on current evidence, neurodegenerative diseases and advanced physiological brain aging appear to us as the most likely involved. The olfactory system plays a determinant role and further attention should be given to these potential mechanisms underlying the robust OI-mortality association. In the future, we should also address the question whether the recovery of olfactory function could have beneficial effects on outcome.

## Author Contributions

VV and CH were the major contributors in writing the manuscript. All authors were involved in manuscript preparation and approved the final version of the manuscript.

## Conflict of Interest

The authors declare that the research was conducted in the absence of any commercial or financial relationships that could be construed as a potential conflict of interest.

## References

[B1] Adams D. R. Kern D. W. Wroblewski K. E. McClintock M. K. Dale W. Pinto J. M. (2018). Olfactory dysfunction predicts subsequent dementia in older U.S. adults. *J. Am. Geriatr. Soc.* 66 140–144. 10.1111/jgs.15048 28944467 PMC6317879

[B2] Ajmani G. S. Suh H. H. Pinto J. M. (2016a). Effects of ambient air pollution exposure on olfaction: a review. *Environ. Health Perspect.* 124 1683–1693. 10.1289/ehp136 27285588 PMC5089874

[B3] Ajmani G. S. Suh H. H. Wroblewski K. E. Kern D. W. Schumm L. P. McClintock M. K. (2016b). Fine particulate matter exposure and olfactory dysfunction among urban-dwelling older US adults. *Environ. Res.* 151 797–803. 10.1016/j.envres.2016.09.012 27692900 PMC5554594

[B4] Al Aïn S. Poupon D. Hétu S. Mercier N. Steffener J. Frasnelli J. (2019). Smell training improves olfactory function and alters brain structure. *Neuroimage* 189 45–54. 10.1016/j.neuroimage.2019.01.008 30630079

[B5] Attems J. Walker L. Jellinger K. A. (2015). Olfaction and aging: a mini-review. *Gerontology* 61 485–490. 10.1159/000381619 25968962

[B6] Berardelli A. Wenning G. K. Antonini A. Berg D. Bloem B. R. Bonifati V. (2013). EFNS/MDS-ES recommendations for the diagnosis of Parkinson’s disease. *Eur. J. Neurol.* 20 16–34. 10.1111/ene.12022 23279440

[B7] Borenstein Graves A. Bowen J. D. Rajaram L. McCormick W. C. McCurry S. M. Schellenberg G. D. (1999). Impaired olfaction as a marker for cognitive decline. Interaction with apolipoprotein E 4 status. *Neurology* 53 1480–1487. 10534255 10.1212/wnl.53.7.1480

[B8] Braak H. Del Tredici K. (2017). Neuropathological staging of brain pathology in sporadic Parkinson’s disease: separating the wheat from the chaff. *J. Parkinsons Dis.* 7 S71–S85. 28282810 10.3233/JPD-179001 PMC5345633

[B9] Calderon-Garciduenas L. Gonzalez-Maciel A. Reynoso-Robles R. Kulesza R. J. Mukherjee P. S. Torres-Jardon R. (2018). Alzheimer’s disease and alpha-synuclein pathology in the olfactory bulbs of infants, children, teens and adults ≤40 years in metropolitan mexico city. *APOE*4 carriers at higher risk of suicide accelerate their olfactory bulb pathology. *Environ. Res.* 166 348–362. 10.1016/j.envres.2018.06.027 29935448

[B10] Cavazzana A. Poletti S. C. Guducu C. Larsson M. Hummel T. (2018). Electro-olfactogram responses before and after aversive olfactory conditioning in humans. *Neuroscience* 373 199–206. 10.1016/j.neuroscience.2018.01.025 29360513

[B11] Cerf-Ducastel B. Murphy C. (2009). Age-related differences in the neural substrates of cross-modal olfactory recognition memory: an fMRI investigation. *Brain Res.* 88 1285–1288. 10.1016/j.brainres.2009.05.086 19505443 PMC2820012

[B12] Conti M. Z. Vicini-Chilovi B. Riva M. Zanetti M. Liberini P. Padovani A. (2013). Odor identification deficit predicts clinical conversion from mild cognitive impairment to dementia due to Alzheimer’s disease. *Arch. Clin. Neuropsychol.* 28 391–399. 10.1093/arclin/act032 23669447

[B13] Correia C. Lopez K. J. Wroblewski K. E. Huisingh-Scheetz M. Kern D. W. Chen R. C. (2016). Global sensory impairment in older adults in the United States. *J. Am. Geriatr. Soc.* 64 306–313. 10.1111/jgs.13955 26889840 PMC4808743

[B14] Croy I. Negoias S. Novakova L. Landis B. N. Hummel T. (2012). Learning about the functions of the olfactory system from people without a sense of smell. *PLoS One* 7:e33365. 10.1371/journal.pone.0033365 22457756 PMC3310072

[B15] Croy I. Nordin S. Hummel T. (2014). Olfactory disorders and quality of life – An updated review. *Chem. Senses* 39 185–194. 10.1093/chemse/bjt072 24429163

[B16] Curtis M. A. Kam M. Nannmark U. Anderson M. F. Zetterstrom Axell M. Wikkelso C. (2007). Human neuroblasts mograte to the olfactory bulb via a lateral ventricular extension. *Science* 315 1243–1249. 10.1126/science.1136281 17303719

[B17] Devanand D. P. (2016). Olfactory identification deficits, cognitive decline, and dementia in older adults. *Am. J. Geriatr. Psychiatry* 24 1151–1157. 10.1016/j.jagp.2016.08.010 27745824 PMC5136312

[B18] Devanand D. P. Lee S. Manly J. Andrews H. Schupf N. Masurkar A. (2015). Olfactory identification deficits and increased mortality in the community. *Ann. Neurol.* 78 401–411. 10.1002/ana.24447 26031760 PMC4546561

[B19] Dong J. Pinto J. M. Guo X. Alonso A. Tranah G. Cauley J. A. (2017). The prevalence of anosmia and associated factors among U.S. black and white older adults. *J. Gerontol. A. Biol. Sci. med. Sci.* 72 1080–1086. 10.1093/gerona/glx081 28498937 PMC5861899

[B20] Doty R. L. (2018). Age-related deficits in taste and smell. *Otolaryngol. Clin. North Am.* 51 815–825. 10.1016/j.otc.2018.03.014 30001793

[B21] Doty R. L. Bromley S. M. (2004). Effects of drugs on olfaction and taste. *Otolaryngol. Clin. N. Am.* 37 1229–1254. 10.1016/j.otc.2004.05.002 15563912

[B22] Doty R. L. Kamath V. (2014). The influences of age on olfaction: a review. *Front. Psychol.* 5:20. 10.3389/fpsyg.2014.00020 24570664 PMC3916729

[B23] Doty R. L. Petersen I. Mensah N. Christensen K. (2011). Genetic and environmental influences on odor identification ability in the very old. *Psychol. Aging* 26 864–871. 10.1037/a0023263 21639645 PMC3631779

[B24] Ekström I. Sjölund S. Nordin S. Nordin Adolfsson A. Adolfsson R. Nilsson L.-G. (2017). Smell loss predicts mortality risk regardless of dementia conversion. *J. Am. Geriatr. Soc.* 65 1238–1243. 10.1111/jgs.14770 28326534

[B25] Fisher D. Li C. M. Chiu M. S. Themann C. L. Petersen H. Jonasson F. (2014). Impairments in hearing and vision impact on mortality in older people: the AGES-Reykjavik Study. *Age. Ageing* 43 69–76. 10.1093/ageing/aft122 23996030 PMC3861337

[B26] Fuller-Thomson E. R. Fuller-Thomson E. G. (2019). Relationship between poor olfaction and mortality. *Ann. Intern. Med.* 171 525–526. 10.7326/L19-0467 31569243

[B27] Garrison J. L. Knight Z. A. (2017). Linking smell to metabolism and aging. *Science* 358 718–719. 10.1126/science.aao5474 29123049 PMC5937531

[B28] Gauthier S. Reisberg B. Zaudig M. Petersen R. C. Ritchie K. Broich K. (2006). Mild cognitive impairment. *Lancet* 367 1262–1270. 16631882 10.1016/S0140-6736(06)68542-5

[B29] Genter M. Doty R. L. (2019). Toxic exposures and the senses of taste and smell. *Handb. Clin. Neurol.* 164 389–408. 10.1016/B978-0-444-63855-7.00022-8 31604559

[B30] Gjerde K. V. Müller B. Skeie G. O. Assmus J. Alves G. Tysnes O.-B. (2018). Hyposmia in a simple smell test is associated with accelerated cognitive decline in early Parkinson’s disease. *Acta Neurol. Scand.* 138 508–514. 10.1111/ane.13003 30058142

[B31] Gopinath B. Anstey K. J. Sue C. M. Kifley A. Mitchell P. (2011). Olfactory impairment in older adults is associated with depressive symptoms and poorer quality of life scores. *Am. J. Geriatr. Psychiatry* 19 830–834. 10.1097/JGP.0b013e318211c205 21422904

[B32] Gopinath B. Russel J. Sue C. M. Flood V. M. Burlutsky G. Mitchell P. (2016). Olfactory impairment in older adults is associated with poorer diet quality over 5 years. *Eur. J. Nutr.* 55 1081–1087. 10.1007/s00394-015-0921-2 25957862

[B33] Gopinath B. Sue C. M. Kifley A. Mitchell P. (2012). The association between olfactory impairment and total mortality in older adults. *J. Gerontol. A. Biol. Sci. Med. Sci.* 67 204–209. 10.1093/gerona/glr165 22080501

[B34] Gouveri E. Katotomichelakis M. Gouveris H. Danielides V. Maltezos E. Papanas N. (2014). Olfactory dysfunction in type 2 diabetes mellitus: an additional manifestation of microvascular disease? *Angiology* 65 869–876. 10.1177/0003319714520956 24554429

[B35] GunzerW. (2017). Changes of olfactory performance during the process of aging – Psychophysical testing and its relevance in the fight against malnutrition. *J. Nutr. Health Aging* 21 1010–1015. 10.1007/s12603-017-0873-8 29083442

[B36] Haehner A. Masala C. Walter S. Reichmann H. Hummel T. (2019). Incidence of Parkinson’s disease in a large patient cohort with idiopathic smell and taste loss. *J. Neurol.* 266 339–345. 10.1007/s00415-018-9135-x 30488238

[B37] Harita M. Miwa T. Shiga H. Yamada K. Sugiyama E. Okabe Y. (2019). Association of olfactory impairment with indexes of sarcopenia and frailty in community-dwelling older adults. *Geriatr. Gerontol. Int.* 19 384–391. 10.1111/ggi.1362 30968523

[B38] Hedner M. Nilsson L.-G. Olofsson J. K. Bergman O. Eriksson E. Nyberg L. (2010). Age-related olfactory decline is associated with the BDNF val66met polymorphism: evidence from a population-based study. *Front. Aging Neurosci.* 2:24. 10.3389/fnagi.2010.00024 20589104 PMC2893376

[B39] Henkin R. I. Schmidt L. Velicu I. (2013). Interleukin 6 in hyposmia. *JAMA Otolaryngol. Head Neck Surg.* 139 728–734. 10.1001/jamaoto.2013.3392 23868430

[B40] Hoffman H. J. Rawal S. Li C.-M. Duffy V. B. (2016). New chemosensory component in the U.S. national health and nutrition examination survey (NHANES): first-year results for measured olfactory dysfunction. *Rev. Endocr. Metab. Disord.* 17 221–240. 10.1007/s11154-016-9364-1 27287364 PMC5033684

[B41] Hoogland J. Post B. de Bie R. M. A. (2019). Overall and disease related mortality in Parkinson’s disease – a longitudinal cohort study. *J. Parkinsons Dis.* 9 767–774. 10.3233/JPD-191652 31498129 PMC6839485

[B42] Huart C. Eloy P. Rombaux P. (2013a). “Olfaction,” in *Nasal Physiology and Pathophysiology of Nasal Disorders*, ed. Önerci T. M. (Berlin: Springer), 113–137.

[B43] Huart C. Rombaux P. Hummel T. (2013b). Plasticity of the human olfactory system: the olfactory bulb. *Molecules* 18 11586–11600. 10.3390/molecules180911586 24048289 PMC6269828

[B44] Huart C. Rombaux P. Hummel T. (2019). Neural plasticity in developing and adult olfactory pathways – focus on the human olfactory bulb. *J. Bioernerg. Biomembr.* 51 77–87. 10.1007/s10863-018-9780-x 30604090

[B45] Hummel T. Henkel S. Negoias S. Galvan J. R. B. Bogdanov V. Hopp P. (2013). Olfactory bulb volume in patients with temporal lobe epilepsy. *J. Neurol.* 260 1004–1008. 10.1007/s00415-012-6741-x 23135292

[B46] Hummel T. Landis B. N. Hüttenbrink K.-B. (2011). Smell and taste disorders. *GMS Curr. Top. Otorhinolaryngol. Head Neck Surg.* 10:Doc04. 10.3205/cto000077 22558054 PMC3341581

[B47] Jung H. J. Shin I.-S. Lee J.-E. (2019). Olfactory function in mild cognitive impairment and Alzheimer’s disease: a meta-analysis. *Laryngoscope* 129 362–369. 10.1002/lary.27399 30565695

[B48] Kim H. Y. Dhong H. J. Min Y. G. Jung Y. G. Chung S. K. (2010). Effects of statins on regeneration of olfactory epithelium. *Am. J. Rhinol. Allergy.* 24 121–125. 10.2500/ajra.2010.24.3455 20338109

[B49] Kim H. Y. Kim J. H. Dhong H. J. Kim K. R. Chung S. K. Chung S. C. (2012). Effects of statins on the recovery of olfactory function in a 3-methylindole-induced anosmia mouse model. *Am. J. Rhinol. Allergy.* 26 e81–e84. 10.2500/ajra.2012.26.3719 22487282

[B50] Kramarow E. Chen L.-H. Hedegaard H. Warner M. (2015). Deaths from unintentional injury among adults aged 65 and over: United States, 2000-2013. *NCHS Data Brief* 199 25973998

[B51] Landis B. N. Konnerth C. G. Hummel T. (2004). A study on the frequency of olfactory dysfunction. *Laryngoscope* 114 1764–1769. 10.1097/00005537-200410000-00017 15454769

[B52] Landis B. N. Hummel T. Hugentobler M. Giger R. Lacroix J. S. (2003). Ratings of overall olfactory function. *Chem. Senses* 28 691–694. 10.1093/chemse/bjg061 14627537

[B53] Laudisio A. Navarini L. Margiotta D. P. E. Fontana D. O. Chiarella I. Spitaleri D. (2019). The association of olfactory dysfunction, frailty, and mortality is mediated by inflammation: result from the InCHIANTI study. *J. Immunol. Res.* 2019:3128231. 10.1155/2019/3128231 30915369 PMC6402210

[B54] Leschak C. J. Eisenberger N. I. (2018). The role of social relationships in the link between olfactory dysfunction and mortality. *PLoS One* 13:e0196708. 10.1371/journal.pone.0196708 29768447 PMC5955501

[B55] Lin H. W. Mahboubi H. Bhattacharyya N. (2019). Hearing difficulty and risk of mortality. *Ann. Otol. Rhinol. Laryngol.* 128 614–618. 10.1177/0003489419834948 30832489

[B56] Liu B. Luo Z. Pinto J. M. Shiroma E. J. Tranah G. J. Wirdefeldt K. (2019). Relationship between poor olfaction and mortality among community-dwelling older adults. *Ann. Intern. Med.* 170 673–681. 10.7326/M18-0775 31035288 PMC7673250

[B57] Lötsch J. Daiker H. Hähner A. Ultsch A. Hummel T. (2015a). Drug-target based cross-sectional analysis of olfactory drug effects. *Eur. J. Clin. Pharmacol.* 71 461–471. 10.1007/s00228-015-1814-2 25666029

[B58] Lötsch J. Geisslinger G. Hummel T. (2012). Sniffing out pharmacology: interactions of drugs with human olfaction. *Trends Pharmacol. Sci.* 33 193–199. 10.1016/j.tips.2012.01.004 22361590

[B59] Lötsch J. Hummel T. (2019). Clinical usefulness of self-rated olfactory performance – A data science-based assessment of 6000 patients. *Chem. Senses* 44 357–364. 10.1093/chemse/bjz029 31077277

[B60] Lötsch J. Knothe C. Lippman C. Ultsch A. Hummel T. Walter C. (2015b). Olfactory drug effects approached from human-derived data. *Drug Discov. Today* 20 1398–1406. 10.1016/j.drudis.2015.06.012 26160059

[B61] Mackay-Sim A. Johnston A. N. B. Owen C. Burne T. H. J. (2006). Olfactory ability in the healthy population: reassessing prebyosmia. *Chem. Senses* 31 763–771. 10.1093/chemse/bjl019 16901951

[B62] Macleod A. D. Taylor K. S. M. Counsell C. E. (2014). Mortality in Parkinson’s disease: a systematic review and meta-analysis. *Mov. Disord.* 29 1615–1622. 10.1002/mds.25898 24821648

[B63] Marin C. Vilas D. Langdon C. Alobid I. Lopez-Chacon M. Haehner A. (2018). Olfactory dysfunction in neurodegenerative diseases. *Curr. Allergy Asthma Rep.* 18:42. 10.1007/s11882-018-0796-4 29904888

[B64] Min J. Y. Min K. B. (2018). Insulin resistance and the increased risk for smell dysfunction in US adults. *Laryngoscope* 129 1992–1996. 10.1002/lary.27093 29330868

[B65] Morales-Medina J. C. Ianitti T. Freeman A. Caldwell H. K. (2017). The olfactory bulbectomized rat as a model of depression: the hippocampal pathway. *Behav. Brain Res.* 317 562–575. 10.1016/j.bbr.2016.09.029 27633561

[B66] Mortreux M. Foppen E. Denis R. G. Montaner M. Kassis N. Denom J. (2019). New roles for prokineticin 2 in feeding behavior, insulin resistance and type 2 diabetes: studies in mice and humans. *Mol. Metab.* 29 182–196. 10.1016/j.molmet.2019.08.016 31668389 PMC6812023

[B67] Negoias S. Pietsch K. Hummel T. (2017). Changes in olfactory bulb volume following lateralized olfactory training. *Brain Imaging Behav.* 11 998–1005. 10.1007/s11682-016-9567-9 27448159

[B68] Oleszkiewicz A. Kunkel F. Larsson M. Hummel T. (in press). Consequences of undetected olfactory loss on human chemosensory communication and well-being. *Prof. Royal Acad. Sci. Phil. Trans. B*. 10.1098/rstb.2019.0265 PMC7209944 32306872

[B69] Oleszkiewicz A. Hummel T. (2019). Whose nose does not know? Demographical characterization of people unaware of ansomia. *Eur. Arch. Otorhinolaryngol.* 276 1849–1852. 10.1007/s00405-019-05414-8 30989334 PMC6529373

[B70] Olofsson J. K. Rönnlund M. Nordin S. Nyberg L. Nilsson L.-G. Larsson M. (2009). Odor identification deficit as a predictor of five-year global cognitive change: interactive effects with age and ApoE-(4). *Behav. Genet.* 39 496–503. 10.1007/s10519-009-9289-5 19633944

[B71] Ottaviano G. Savietto E. Scarpa B. Bertocco A. Maculan P. Sergi G. (2018). Influence of number of drugs on olfaction in the elderly. *Rhinology* 56 351–357. 10.4193/Rhin17.152 29938715

[B72] Palouzier-Paulignan B. Lacroix M. C. Aimé P. Baly C. Caillol M. Congar P. (2012). Olfaction under metabolic influences. *Chem. Senses* 37 769–797. 10.1093/chemse/bjs059 22832483 PMC3529618

[B73] Pence T. S. Reiter E. R. DiNardo L. J. Costanzo R. M. (2014). Risk factors for hazardous events in olfactory-impaired patients. *JAMA Otolayrngol. Head Neck Surg.* 140 951–955. 10.1001/jamaoto.2014.1675 25170573

[B74] Pinto J. M. Wroblewski K. E. Huisingh-Scheetz M. Correia C. Lopez K. J. Chen R. C. (2017). Global sensory impairment predicts morbidity and mortality in older U.S. *adults*. *J. Am. Geriatr. Soc.* 65 2587–2595. 10.1111/jgs.15031 28942611 PMC6317884

[B75] Pinto J. M. Wroblewski K. E. Kern D. W. Schumm L. P. McClintock M. K. (2014). Olfactory dysfunction predicts 5-year mortality in older adults. *PLoS One* 9:e107541. 10.1371/journal.pone.0107541 25271633 PMC4182669

[B76] Reichert J. L. Schöpf V. (2018). Olfactory loss and regain: lessons for neuroplasticity. *Neuroscientist* 24 22–35. 10.1177/1073858417703910 28459173

[B77] Richardson B. E. Vander Woude E. A. Sudan R. Thompson J. S. Leopold D. A. (2004). Altered olfactory acuity in the morbidly obese. *Obes. Surg.* 7 967–969. 10.1381/0960892041719617 15329187

[B78] Rombaux P. Mouraux A. Bertrand B. Nicolas G. Duprez T. Hummel T. (2006a). Olfactory function and olfactory bulb volume in patients with postinfectious olfactory loss. *Laryngoscope* 116 436–439. 10.1097/01.mlg.0000195291.36641.1e 16540905

[B79] Rombaux P. Mouraux A. Bertrand B. Nicolas G. Duprez T. Hummel T. (2006b). Retronasal and orthonasal olfactory function in relation to olfactory bulb volume in patients with posttraumatic loss of smell. *Laryngosope* 116 901–905. 10.1097/01.mlg.0000217533.60311.e7 16735894

[B80] Rottstädt F. Han P. Weidner K. Schellong J. Wolff-Stephan S. Strauß T. (2018). Reduced olfactory bulb in depression – A structural moderator analysis. *Hum. Brain Mapp.* 39 2573–2582. 10.1002/hbm.24024 29493048 PMC6866619

[B81] Sanford A. M. (2017). Mild cognitive impairment. *Clin. Geriatr. Med.* 33 325–337. 10.1016/j.cger.2017.02.005 28689566

[B82] Santos D. V. Reiter E. R. DiNardo L. J. Costanzo R. M. (2004). Hazardous events associated with impaired olfactory function. *Arch. Otolaryngol. Head Neck Surg.* 130 317–319. 15023839 10.1001/archotol.130.3.317

[B83] Schubert C. R. Cruickshanks K. J. Fischer M. E. Huang G.-H. Klein B. E. K. Klein R. (2012). Olfactory impairment in an adult population: the beaver dam offspring study. *Chem. Senses* 37 325–334. 10.1093/chemse/bjr102 22045704 PMC3325763

[B84] Schubert C. R. Cruickshanks K. J. Fischer M. E. Huang G.-H. Klein R. Tsai M. Y. (2015a). Carotid intima media thickness, atherosclerosis, and 5-year decline in odor identification: the beaver dam offspring study. *J. Geron. A. Biol. Sci. Med. Sci.* 70 879–884. 10.1093/gerona/glu15 25182599 PMC4481687

[B85] Schubert C. R. Cruickshanks K. J. Fischer M. E. Klein E. K. Klein R. Pinto A. A. (2015b). Inflammatory and vascular markers and olfactory impairment in older adults. *Age. Ageing* 44 878–882. 10.1093/ageing/afv075 26082178 PMC4547926

[B86] Schubert C. R. Cruickshanks K. J. Klein B. E. K. Klein R. Nondahl D. M. (2011). Olfactory impairment in older adults: 5-year incidence and risk factors. *Laryngoscope* 121 873–878. 10.1002/lary.21416 21298645 PMC3063862

[B87] Schubert C. R. Fischer M. E. Pinto A. A. Klein B. E. K. Klein R. Tweed T. S. (2016). Sensory impairments and risk of mortality in older adults. *J. Gerontol. A. Biol. Sci. Med. Sci.* 72 710–715. 10.1093/gerona/glw036 26946102 PMC5861964

[B88] Sivam A. Wroblewski K. E. Alkorta-Aranburu G. Barnes L. L. Wilson R. S. Bennett D. A. (2016). Olfactory dysfunction in older adults is associated with feelings of depression and loneliness. *Chem. Senses* 41 293–299. 10.1093/chemse/bjv088 26809485 PMC5006107

[B89] SomekawaS.MineT.OnoK.HayashiN.ObuchiS.YoshidaH. (2017). Relationship between sensory perception and frailty in a community-dwelling elderly population. *J. Nutr. Health Aging* 21 710–714. 10.1007/s12603-016-0836-5 28537337

[B90] Sorokowska A. Drechsler E. Karwowski M. Hummel T. (2017). Effects of olfactory training: a meta-analysis. *Rhinology* 55 17–26. 10.4193/Rhin16.195 28040824

[B91] Stevenson R. J. (2010). An initial evaluation of the functions of human olfaction. *Chem. Senses* 35 3–20. 10.1093/chemse/bjp083 19942579

[B92] Taylor C. A. Greenlund S. E. McGuire L. C. Lu H. Croft J. B. (2017). Deaths from Alzheimer’s disease – United States, 1999-2014. *MMWR Morb. Mortal. Wkly. Rep.* 66 521–526. 10.15585/mmwr.mm6620a1 28542120 PMC5657871

[B93] Wang Q.-S. Tian L. Huang Y.-L. Qin S. He L.-Q. Zhou J.-N. (2002). Olfactory identification and apolipoprotein E (4 allele in mild cognitive impairment. *Brain Res.* 951 77–81. 10.1016/s0006-8993(02)03137-2 12231459

[B94] Wegener B.-A. Croy I. Hähner A. Hummel T. (2018). Olfactory training with older people. *Int. J. Geriatr. Psychiatry* 33 212–220. 10.1002/gps.4725 28429377

[B95] Wilson R. S. Arnold S. E. Schneider J. A. Boyle P. A. Buchman A. S. Bennett D. A. (2009). Olfactory impairment in presymptomatic Alzheimer’s disease. *Ann. N. Y. Acad. Sci.* 1170 730–735. 10.1111/j.1749-6632.2009.04013.x 19686220 PMC2857767

[B96] Wilson R. S. Yu L. Bennett D. A. (2011). Odor identification and mortality in old age. *Chem. Senses* 36 63–67. 10.1093/chemse/bjq098 20923931 PMC3002399

[B97] Yaldizi O. Penner I.-K. Yonekawa T. Naegelin Y. Kuhle J. Pardini M. (2016). The association between olfactory bulb volume, cognitive dysfunction, physical disability and depression in multiple sclerosis. *Eur. J. Neurol.* 23 510–519. 10.1111/ene.12891 26699999

[B98] Yulug B. Saatci O. Isiklar A. Hanoglu L. Kilic U. Ozansoy M. (2019). The association between HbA1c levels, olfactory memory and cognition in normal, pre-diabetic and diabetic persons. *Endocr. Metab. Immune Disord. Drug Targets.* 10.2174/1871530319666190614121738 [Epub ahead of print], 31203811

[B99] Zou L.-Q. Yang Z.-Y. Wang Y. Lui S. S. Y. Chen A.-T. Cheung E. F. C. (2016). What does the nose know? Olfactory function predicts social network size in human. *Sci. Rep.* 6:25026. 10.1038/srep25026 27109506 PMC4842975

